# Snake-Efficient Feature Selection-Based Framework for Precise Early Detection of Chronic Kidney Disease

**DOI:** 10.3390/diagnostics13152501

**Published:** 2023-07-27

**Authors:** Walaa N. Ismail

**Affiliations:** Department of Management Information Systems, College of Business Administration, Al Yamamah University, Riyadh 11512, Saudi Arabia; w_abdelfattah@yu.edu.sa

**Keywords:** chronic kidney disease, convolution neural networks, machine learning, snake optimization, feature selection, medical data analysis, deep neural network

## Abstract

Chronic kidney disease (CKD) refers to impairment of the kidneys that may worsen over time. Early detection of CKD is crucial for saving millions of lives. As a result, several studies are currently focused on developing computer-aided systems to detect CKD in its early stages. Manual screening is time-consuming and subject to personal judgment. Therefore, methods based on machine learning (ML) and automatic feature selection are used to support graders. The goal of feature selection is to identify the most relevant and informative subset of features in a given dataset. This approach helps mitigate the curse of dimensionality, reduce dimensionality, and enhance model performance. The use of natural-inspired optimization algorithms has been widely adopted to develop appropriate representations of complex problems by conducting a blackbox optimization process without explicitly formulating mathematical formulations. Recently, snake optimization algorithms have been developed to identify optimal or near-optimal solutions to difficult problems by mimicking the behavior of snakes during hunting. The objective of this paper is to develop a novel snake-optimized framework named CKD-SO for CKD data analysis. To select and classify the most suitable medical data, five machine learning algorithms are deployed, along with the snake optimization (SO) algorithm, to create an extremely accurate prediction of kidney and liver disease. The end result is a model that can detect CKD with 99.7% accuracy. These results contribute to our understanding of the medical data preparation pipeline. Furthermore, implementing this method will enable health systems to achieve effective CKD prevention by providing early interventions that reduce the high burden of CKD-related diseases and mortality.

## 1. Introduction

Almost 800 million people around the world are estimated to have chronic kidney disease (CKD). Waste products and excess water are filtered by the kidneys and excreted through the urine. As CKD progresses, the body may accumulate excessive amounts of fluids, electrolytes, and waste products [1,2]. End-stage renal disease (ESRD) is characterized by kidney failure. The term chronic kidney disease (CKD) refers to a condition in which the kidneys are no longer able to filter blood as effectively as they should [1,2]. There is no doubt that CKD can affect anyone, however, some people are more susceptible to it than others. Specifically, patients who are suffering from heart disease, diabetes, old age, abnormal calcium levels, or abnormal potassium levels. The World Health Organization reported that there is an expected increase in the prevalence of CKD among adults aged 30 years or older from 13.2% in 1999–2010 to 16.7% in 2030 [3]. In addition, CKD is associated with an increased risk of cardiovascular disease. In certain circumstances, dialysis or a transplant may be necessary. It is important to note that although chronic kidney disease (CKD) is a serious medical condition, it can be treated if it is detected early enough.

Deep learning (DL) has been widely used in recent years for the analysis of medical data and the recognition of patterns in those data [4,5,6]. In deep learning, computers are capable of receiving new information, decoding data, and processing the output without the assistance of a human. Several aspects of this field could have potential impacts on future computer technologies, including biomedical signals, image processing, and pattern recognition [7,8,9]. For these approaches, an unstructured set of data is provided so that computers can learn how to recognize ideal patterns related to a specific problem domain. Although deep learning can perform many of the tasks performed by machine learning, its functions are very different. First, the amount of data that can be absorbed by machine learning is known to be limited. In addition, deep learning methods do not require manual extraction of features; instead, they use neural networks to learn these features directly from data. There are three main components to DL models: the data it uses, the network design, and the training parameters. In order to achieve maximum efficiency, deep learning models must achieve the perfect balance between these three elements [10]. Various conventional deep neural network (CNN) techniques have been applied to diagnose CKD in its initial stages after several trials to confirm the design of the model. To improve the accuracy of the statistical analysis, Acharya et al. reviewed the data set of medically related diseases. Various machine learning methods (such as CNN) are used in CKD image analysis to achieve 94% classification accuracy [11]. Nithya et al. created a method for classifying and grouping the data as part of their investigation of the renal disease data set [12]. The authors gathered the most recognizable photographs from various collections of photos using the K-Means clustering method. Researchers used artificial neural networks to estimate the prediction of kidney diseases to calculate the training accuracy of 99.61% [13]. Navaneeth and Suchetha [14] devised a strategy to predict chronic kidney diseases using CNN and SVM. The authors believe that the sensitivity, specificity, and accuracy of the prediction are higher. Bevilacqua et al. used frameworks and methods to assess chronic kidney disease [15]. The authors estimated that the disease data set was classified with 85% precision using CNN machine learning technology. Most previous studies have had difficulty obtaining high testing accuracy when using a small training dataset. Several previous studies have been plagued by overfitting problems caused by the use of different datasets, or they have reported accuracy, but some performance adjustments are still missing (i.e., without clearly justifying if the obtained accuracy is for training or testing). Additionally, working with noisy or blurred data can be challenging. Missing values have been handled by researchers using the mean and median method, but this method is not ideal for medical data sets, as the value of the missing value may be higher or lower than the mean and median.

Deep neural networks rely heavily on optimization algorithms for a number of purposes, including the extraction of features, the selection of layers, and the tuning of hyperparameters [16,17]. In order to extract features from raw data, automatic learning is used to identify relevant representations or features. The learned features allow the neural network to make accurate predictions or classifications of the data by capturing important patterns, structures, and characteristics. A multidisciplinary approach is required to make an accurate diagnosis of CKD predisposing during the early stages. As a result, assessing predisposing factors can be challenging because it requires consideration of a number of conditions that are intricately related and where the data are incomplete, contradictory, or unavailable. To overcome the complexity of CKD data, snake optimization (SO) algorithms [18,19] can be utilized to automatically learn a hierarchy of CKD features. As a result of this feature extraction capability, deep learning eliminates the need for manual feature engineering, which is time-consuming and prone to errors.

The goal of this paper is to propose a novel snake-optimized framework named CKD-SO for CKD data analysis. As a result of the present study, the following novel contributions have been made:

1. Provide a novel framework CKD-SO for the analysis of CKD data using two novel custom CNN architectures. The architectures were constructed from scratch and trained so that they were capable of identifying kidney disease accurately.
2. An efficient and reliable pipeline for the preparation of medical data is presented, thus improving the learnability of the devised model and enhancing its prediction accuracy.
3. After the pre-processing stage, the snake optimization algorithm is used to select the optimal combination of CKD features automatically, and machine learning algorithms are utilized to predict CKD from the collected features, resulting in high accuracy and reduced likelihood of incorrect diagnoses.

This paper is organized as follows: Section 2 provides background information regarding the proposed algorithm. The proposed algorithm is detailed in Section 3. Section 4 and Section 5 show the experimental design and numerical results needed to evaluate the proposed algorithm. A summary of our conclusions and future work is provided in Section 6.

## 2. Related Work

Traditional machine learning approaches and deep learning approaches are the most common approaches used to detect CKD [20,21]. It is generally accepted that traditional machine learning approaches consist of a two-stage process in which medical information is extracted and then classification algorithms are developed to detect CKD. Krishnamurthy et al. developed a machine-learning model based on data from patients who suffered from more than one disease at the same time [22]. As a result of a 5-fold cross-validation procedure used to assess performance metrics, CNN performed well based on a data set derived from Taiwan’s National Health Insurance Research Database. Multiple classifiers were used in the analysis of the dataset, including convergent neural networks (CNN). In an additional study [23] a machine learning-based approach to the diagnosis of chronic kidney disease was used. A combination of optimal subset regression and RF was used to extract features. After applying four machine learning algorithms, they achieved an accuracy of 100% using the random forest method. As described in [24], the authors used three machine-learning techniques to diagnose CKD: logistic regression, wide and deep learning, and FNN. Additionally, the mean median method was used to handle missing data, the Min Max Scaler was used to normalize the data, and the SMOTE method was used to oversample the data. The FNN achieved an AUC score of 0.99 for both real and oversampled data. Using RBF kernels, the authors of another study applied SVM to categorize patients with CKD from patients without CKD. Six criteria were combined to achieve an overall classification precision of 94.44%. In [25], the authors propose a method that relies on artificial classification techniques to detect kidney disease. The SVM sensitivity, specificity, and accuracy metrics were used to determine the best results. Yashfi [26] proposed to analyze the data of CKD using random forests and artificial neural networks. As a result, 20 out of 25 features have been extracted with a high level of 97.12% accuracy.

An ensemble of deep learning-based clinical decision support systems (EDL-CDSS) was presented by Alsuhibany et al. [27] for the diagnosis of chronic kidney disease in an IoT environment. A different approach is employed to detect outliers, which utilizes the Adaptive Synthetic (ADASYN) technique and employs an ensemble of three models, namely the deep belief network (DBN), the kernel extreme learning machine (KELM), and the convolutional neural network with gated recurrent networks. unit (CNN-GRU). DBN and CNN-GRU hyperparameters are also tuned using a quasi-oppositional butterfly optimization algorithm (QOBOA).

In [28], cross-validation of K-structured K-folds was used to validate ML models, SMOTE was used to manage data imbalances, the Local Outlier Factor (LOF) was used to remove outliers, the KNN computation was used to calculate missing values and a new hybrid feature selection method was used to eliminate redundant features. A random forest classifier was found to be 100% accurate in predicting CKD without data leakage among the eight classifiers used in this research.

In addition to supervised machine learning models, high-performance neural networks driven by nonlinear AI, such as SOFNN-HPS (Self-Organizing Fuzzy Neural Network with Hybrid Particle Swarm Optimization) and GK-ARFNN (Gaussian Kernel-Based Adaptive Resonance Fuzzy Neural Network), demonstrate their ability to analyze medical data [29,30]. SOFNN-HPS is a hybrid neural network that combines fuzzy logic with neural networks to create models that can handle complex data. It optimizes the parameters of the model using particle swarm optimization algorithms. GK-ARFNN is also a hybrid neural network that combines fuzzy logic and neural networks. However, it uses an optimization algorithm that is different from SOFNN-HPS. GK-ARFNN uses a combination of Gauss kernel functions and adaptive resonance theory (ART) to create models that can adapt to changing data inputs. In terms of performance, both models have shown good results in the diagnosis and prediction of medical diseases. However, the choice between the two models may depend on the specific requirements of the application. SOFNN-HPS is preferred for applications that require high-level parameter optimization, while GK-ARFNN is preferred for applications that need adaptability to change data input. In [31], an optimal set of features is used to develop a decision-making system for improving CKD prediction performance. Pretrained deep learning models are integrated with support vector machines (SVMs) as metalearner models in the proposed ensemble model. For the purpose of selecting the optimal feature list, four methods of feature selection are employed. The proposed Layer2 scores 99.69%, 99.71%, 99.69%, and 99.69%, respectively, for accuracy, precision, recall, and F1.

An intelligent classification and prediction method for chronic kidney disease (CKD), was published in [32]. In this study, it is shown that there are a total of 24 characteristics, and some of these features have been selected using DFS. A comparison of three classification algorithms is taken into account, including genetic algorithms (GA), adaptive classification (AC), and particle swarm optimization (PSO). According to the results of the four algorithms, the accuracy was 95.00, 87.50, 85.00, and 75.00 for D-ACO, PSO, AC, and GA, respectively. From Table 1, the following research gaps were identified:

- Most traditional feature selection methods use fixed evaluation criteria or scoring functions to assess the quality of feature subsets. In CKD detection, these criteria may not adequately capture the complex relationships and interactions between CKD features. The present study addresses the issue of fixed feature selection by automatically selecting features’ combinations with the help of the SO algorithm.
- The majority of research to date has focused on the application of CNN for the enhancement of image data sets to achieve the desired results, with only a few publications using CNN to detect CKD from medical records. This leads to a loss of the potential benefits of CNN in terms of classification and identification of the most important features automatically without the intervention of a human. Due to the great ability of neural models to handle nonlinearity in data, this study employed two custom CNN models that are able to adapt to the crucial CKD information through layers of neurons that are present in the structure independently.
- The accuracy of the models obtained for the training or testing phases has not been formalized. This implies that no discussion is provided about the type of accuracy obtained. Thus, imputed accuracy values in such models are susceptible to deviating from the overall tendency to be accurate. The testing and training accuracy of the devised models is effectively justified through the use of a variety of evaluation metrics.
- Most of the literature has trained models using unbalanced data, which produced biased results. Additionally, several studies did not consider noisy or blurred data, which can lead to the appearance of outliers in numerical features.A dedicated pipeline has been utilized in the present study to handle unbalanced and missing data.

## 3. Proposed Framework

**Figure 1:**
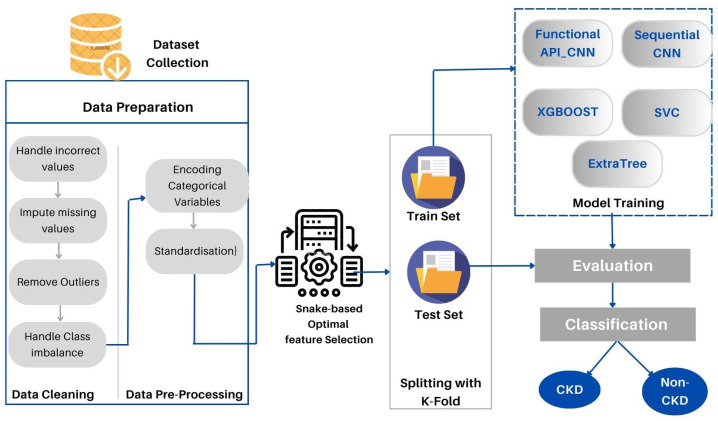
The proposed CKD-SO architecture.

This section provides a detailed description of the proposed method. Furthermore, the data set used is described in detail. As part of the proposed approach, CKD medical data are directly loaded into the deep modeling framework to obtain the final decision. CKD-SO involves training a system that is potentially complicated using a single model that encompasses the whole target system. Figure 1 represents the block diagram of the designed system. First, we collected the data sets and prepared them for analysis. In the following step, the data is divided into two groups: training and testing sets. To avoid data leakage and overloading, we first implement data preparation techniques in training sets and apply them to test sets. Categorical characteristics are encoded and missing values are imputed. The next step is to remove outliers and balance the data classes. The proposed method learns features and selects the most appropriate features by using snake optimization algorithms. The Auto-Keras based-CNN was used to develop two new models to classify CKD data. As a final step, the devised models are tested and evaluated in terms of their performance.

### 3.1. CKD Data Input Layer

In order to create a predictive model using machine learning algorithms, it is necessary to simulate real-life data that have never been seen before and determine the best way to predict or categorize it. Models that suffer from data leakage cannot operate efficiently when faced with new data in the real world as a result.

We collected a dataset (as shown in Table 2) from the Machine Learning Repository at the University of California, Irvine. There are 400 samples, and many missing values, extraneous, typing errors, class imbalances, etc. Table 3 represents a statistical report on the state of the data, which includes many important points such as the status of missing values, distribution ratios between each category of data, and information on the balance of the data. In the following subsections, the exact details of data preparation and preprocessing will be described in detail.

#### 3.1.1. Data Preparation

The first step in preventing data leakage is to prepare the data records with the following processes:

1. Handle incorrect values.
2. Impute missing values.
3. Remove outliers.
4. Handle class imbalance.

**Handling incorrect values:** A total of 400 samples were analyzed from the collected dataset, 250 of which were CKD samples and 150 of which were NOTCKD samples. Due to typographical errors, the functions of the plethora of packed cells (PCV), the number of red cells (RC), and the number of white cells (WC) were incorrectly considered nominal. The data types representing these properties are converted to numerical data types. The Python map method has also been used to correct a few other typos in other features.

categorical_cols are:  
[‘rbc’,‘pc’,‘pcc’,‘ba’,‘htn’,‘dm’,‘cad’,‘appet’,‘pe’,‘ane’]Numeric_cols are:  [‘age’,‘bp’,‘sg’al’,‘su’,‘bgr’,‘bu’,‘sc’sod’pot’hemo’,‘pcv’,

‘wc’,‘rc’,‘classification’]



**Figure 2:**
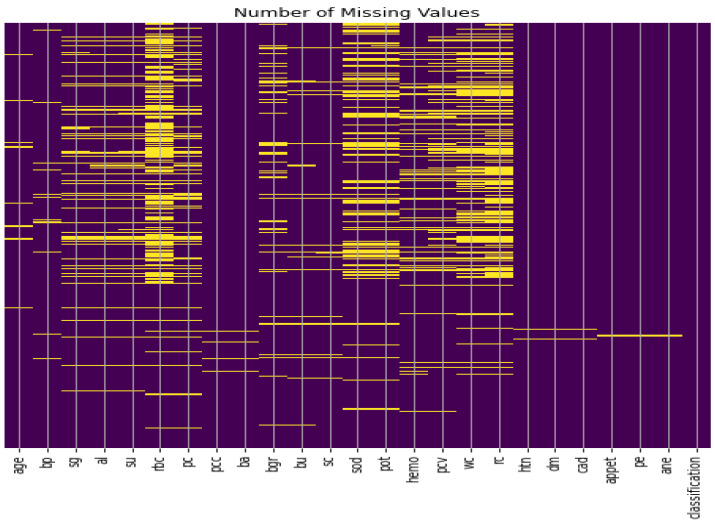
CKD dataset missing values.

**Missing values imputation:** The data set consisting of 24 features and contains 1008 missing values, as illustrated in Figure 2. Therefore, handling such missing values is a very difficult decision to make, especially when working with medical data and attempting to build a model that generates real results. ’Bfill’ is used to handle missing values in categorical variables, and ’mean’ method is used to handle missing values in numerical variables [39]. Furthermore, we applied the compensation for lost values method. The compensation for lost values method is a technique used to estimate missing values based on the values of other variables in the dataset [39].

**Outliers Removal:** Machine learning models are adversely affected by outliers. This allows a model to be formed on irrelevant data. The authors of [40] pointed out that if external detection is implemented effectively, machine learning models for the analysis of medical data will produce more accurate results and diseases will be detected early. We have applied the local outlier factor (LOF) technique to remove outliers based on data sets and problem statements in this study. The statistical and distance-based outlier detection methods are more efficient than the density-based methods. A population’s location is determined by a comparison between the neighboring cities K and the calculated density. Extroversions are extracted from samples by comparing the density of the localities with the density of the neighboring k. Based on 20 neighboring samples, we calculated the Euclidean distance and discovered around 26 samples, including deviations. With the training data set removed, we are left with approximately 274 samples. we have selected several important columns, on the basis of which we will filter the data based on their values. There are five columns that we have selected: ’age’, ’hemo’, ’pcv’, ‘rc’, and ‘sg’. In the target column, these columns are among the most influential columns. Therefore, we will eliminate their outliers and then focus on such data. Second, duplicate values can result in deviations or biases from the model to specific values as a result of either their large size and their effect on the target column or their repetition and thus their formation as a block that attracts the model to it. The model is therefore biased towards one data type rather than the other as a result, and any duplicate values must therefore be eliminated if they are found.

**Handling class imbalance:** Machine learning models are affected by class imbalances in almost every domain in which real-world data is available. Machine learning models perform poorly when dealing with a small number of data classes [41]. A model that is biased toward a specific category of data will produce an algorithm that performs well with a specific category of data and performs poorly on other categories. There are several ways to avoid this problem, including collecting data from its primary sources, balancing data using dedicated algorithms for that purpose, or developing a universal algorithm. Our data has already been collected, so we will not use this solution; According to [42], when there are many samples in a data set, undersampling may be a good method for managing data imbalances. However, when the data set is small, the synthetic oversampling technique is most suitable. In our research, a synthetic minority oversampling technique (SMOTE [43]) was used. A SMOTE method increases the representation of minority classes in a dataset by generating synthetic samples. As a result, the issue of imbalance is mitigated, and a more balanced dataset is provided to the classifier for learning purposes. Instead of simply duplicating existing samples, synthetic samples resemble the minority class, helping to capture the underlying patterns and improve classification. The use of this method will also help to overcome the problem of overfitting caused by random over-sampling. Due to the elimination of deviations, there were approximately 274 samples, 114 of which had CKD, and 184 were not affected.

#### 3.1.2. Data Pre-Processing

The following section describes the different stages of data preprocessing.

**Encoding Categorical Variables:** The purpose of this phase is to convert categorical values into numerical values so that machine learning algorithms can correctly recognize and understand them. The data set cleaning generated ten categories of features (names). The value of these categories is encoded as 0 or 1 using a hot encoding technique.

**Standardization:** The last step before starting to build a predictive model is to ensure that all properties are measured at the same level by using data standardization. The main advantage of this method is to improve the performance of model learning and classification. We used Scikit-learn [16] to standardize the features using Equation (1) a sample *x* with a standard value is calculated. The mean of the training sample is μ and the standard deviation is σ.

(1) $$z=\frac{x-\mu }{\sigma }$$

Z-scores are calculated by dividing a particular value by the number of standard deviations it deviates from the mean. The positive z-score denotes a value above the mean, whereas the negative z-score denotes a value below the mean [44].

### 3.2. Feature Selection Layer

**Figure 3:**
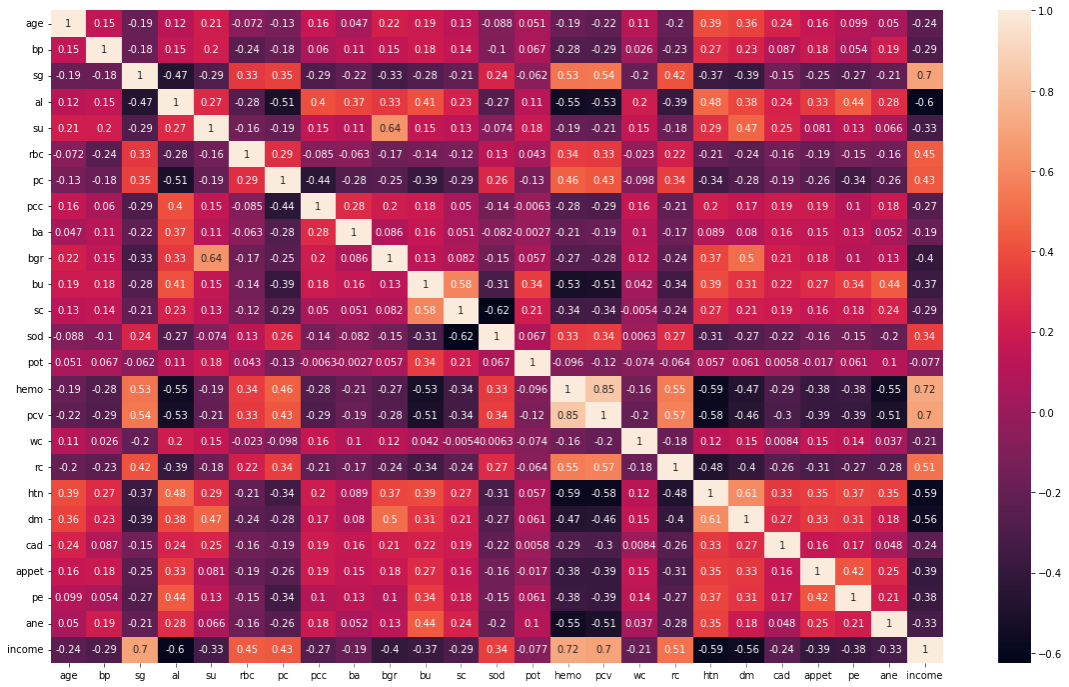
Correlation coefficient of CKD dataset.

**Figure 4:**
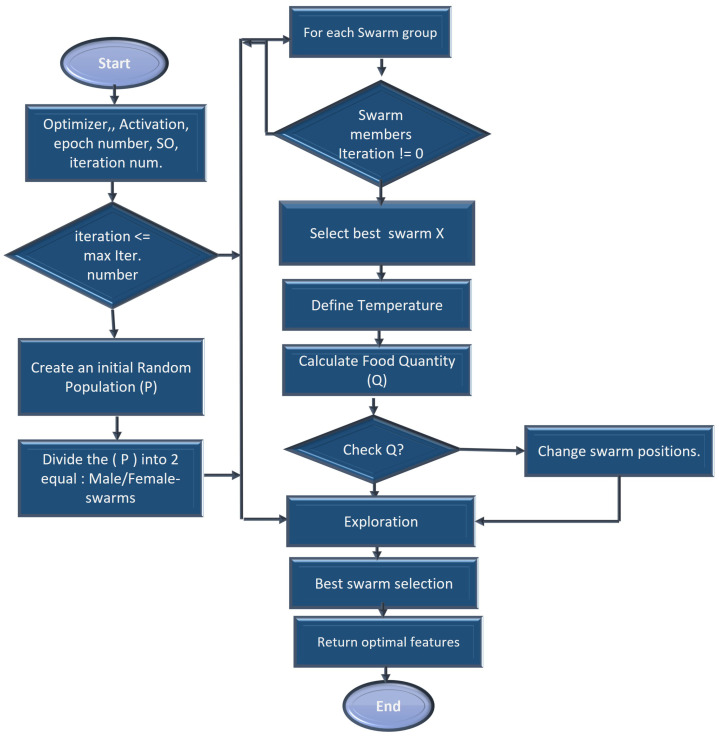
Snake-based optimal CKD feature selection process.

A feature extraction and selection process aims to identify those features from the original dataset which are most relevant to the task at hand and to discard those features that are not relevant or redundant. A machine learning model should benefit from the selected features by improving its performance and reducing the complexity of computation. To gain a better understanding of the data, one can look for correlations between the features and the target. The correlation coefficient is not the most effective method of evaluating the “relevance” of a feature, but it does provide us with some insight into possible relationships within the data. The heat map is shown in Figure 3 indicates that there are some features that have a high correlation coefficient, but there are also some features that show a strong decrease in the correlation coefficient. To extract and select optimal features combinations from CKD data, the snake optimization algorithm (SOA) is utilized in CKD-So (as shown in Figure 4). Based on snake behavior, such as their hunting and movement patterns, this model was developed. An overview of how the snake optimization algorithm can be used to extract and select features is provided below:

As a start, snake optimization is based on an unplanned population in which each snake represents a subset of potential features, each with a binary value (selected or not selected).

An initialization step using all CKD collected features is performed in the function in order to determine the snakes’ position, food quantity, and available temperature, as well as the location of the male/female snakes. Afterward, modernization is carried out through the phases of exploration and exploitation. The exploration phase is characterized by a lack of adequate food. Therefore, the snake is searching for food at a variety of locations. Initialize the location of swarms in the search space *D* using rand value ∈ [0, 1] between min and max values for the data.

(2) $$swarm_{i}=swarm_{min}+rand\times \left(swarm_{max}-swarm_{min}\right).$$

assign the environments’ temperature and quantity of food following Equations (3) and (4).

(3) $$Quantity\left(F\right)=C\times Exp\left(\frac{n-N}{N}\right)$$

where *C* is the constant value equals 0.5, *N* is the total number of iterations and *n* is the current iteration.

(4) $$T=exp\left(\frac{-n}{N}\right)$$

afterward, segment the population size Num into two equal sets of male/female groups $S_{Female}$ and $S_{male}$.

(5) $$S_{male}≃S_{female}=\frac{Num}{2}$$

explore the search space (food not found) to obtain the best candidate for each group of female and male swarms and the location of food in the exploration phase, the next position of the male and female is assigned based on ambient temperature (T).

Food quantity *Quantity*(*F*) is calculated using the below equation:

In the following equation, the new positions $J^{th}$ of the male and female are represented when the temperature rises above the threshold.

(6) $$Y_{j,s}^{n+1}=Y_{\left(rand,s\right)}^{n}\pm D\times B_{s}\times \left(\left(Y_{max}-Y_{min}\right)\times rand+Y_{min}\right)$$

where rand represents the random position of the male and female. *D* is constant, D=0.05. This rand function mimics the movement of snakes when searching for food by incorporating the concept of local and global search. A local search enables the refinement of feature subsets within a local neighborhood, whereas a global search allows exploration across various regions of the feature space and may reveal improved feature combinations.

As a result, the new position of the fighting mode can be calculated using the following equations:

(7) $$Y_{j,s}^{n+1}=Y_{\left(j,s\right)}^{n}\pm D\times MF\times rand\times \left(Y_{\left(optimal,j\right)}-Y_{\left(j,s\right)}^{n}\right))$$

(8) $$Y_{j,g}^{n+1}=Y_{\left(j,g\right)}^{n}\pm D\times FF\times rand\times \left(Y_{\left(optimal,s\right)}-Y_{\left(j,g\right)}^{n}\right))$$

where the fighting capability of the female is represented by FF, the fighting capability of the male is represented by MF. A female’s mating capacity is represented by $S_{j}$ and a male’s mating capacity is represented by $S_{s}$. Optimal positions of swarm individuals are represented as $Y_{\left(rand,s\right)}^{n}$, and D=2.

It is reasonable to assume that during the process of modernization, the number of males and females will be the same. Until the maximum number of iterations *N* has been reached, this modernization process is continued. Each snake in the population should be evaluated based on its fitness using an optimization function SO. The fitness function measures the quality or effectiveness of CKD-selected features. SO is a function that implements the SOA algorithm. This function optimizes an objective function, $Fitness(Y_{j,x}^{n+1})$, given a set of inputs.

(9) $$Fitness\left(Y_{j,x}^{n+1}\right)=exp\left(-\frac{g_{rand}}{x_{j,x}}\right)$$

where *x* represents a male or female snake solution. Once the optimization process has been completed, select the optimal snake combination $Optimal_{CKD}$ whose fitness is the highest as the final feature subset. A subset $Optimal_{CKD}$ represents the features that will be used for subsequent machine learning tasks.

### 3.3. Data Classification Layer

For the detection of CKD from health records, three machine learning algorithms including XGBOOSt, ExtraTree, and SVM are utilized. Additionally, two new customized CNN-based models were used. The details of each model are given in the following subsections.

#### 3.3.1. Functional API_CNN Model

This network was built on the basis/method of the Keras Functional API, and this method has many advantages, including that it allows you to connect layers in the network with other layers, regardless of the location of that layer in the network, in addition to providing developers with many important features in controlling the flow of data through layers in the artificial network. This helps us form and build complex and interlocking artificial networks to solve difficult problems and discover difficult patterns in the data, and thus obtain a better and more reliable level of accuracy. This architecture has a first layer, the input layer, which is not considered an actual layer in likeness since it does not perform any processing on the data entering through it. It contains 24 data entry positions, which correspond to how many columns or features there are in the data. In the second layer, which is Dense type, there are 32 units or neurons.

**Figure 5:**
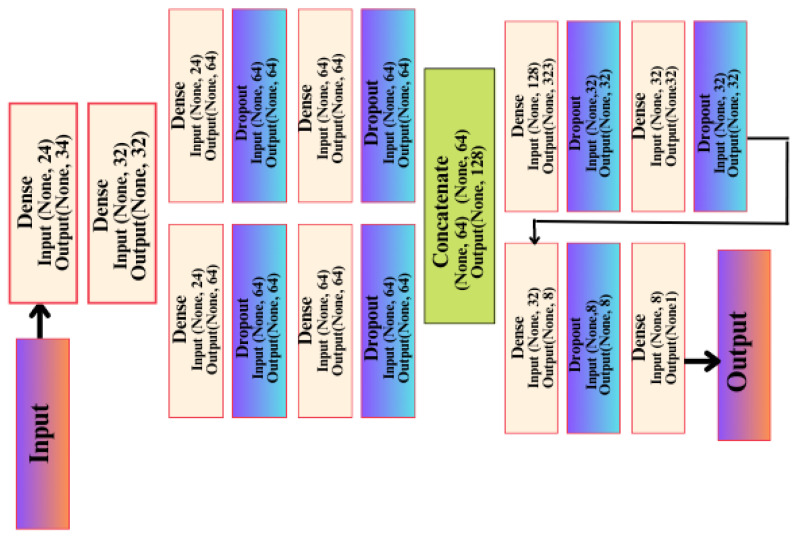
API-CNN model architecture.

These units receive data from the input layer and perform a variety of calculations and processing on it. As with the second layer, the third layer, dense, also repeats the same process; however, it focuses on learning different features that allow the artificial network to discover patterns in data or relationships between inputs and outputs, resulting in 1056 trainable parameters being generated from this layer. Afterward, the artificial network begins to divide into right and left paths. On the right side of the model, the fifth layer is a dropout layer with a random loss rate of 0.01, and its function is explained by neglecting some of the trainable parameters at random, reducing overfitting through this layer from the saving of features derived from the upper layers. The reason for this is that each time the data is entered that layer provides a different set of learnable parameters for the next layer, so good learning takes place and more features are extracted in each layer. The left path contains four layers with the same arrangement and coordination as the right path, as well as the same number of trainable parameters as the right path. It is therefore nothing more than a re-creation of the layers, but it has a different aspect of an artificial network, namely the random process of dropping out layers as well as learning the features with random weights at the beginning of the training process. This is carried out randomly and in a different manner for each layer and each track. A final compiling stage of the API-CNN network was used for a total of 19,857 trainable parameters, which makes use of the SoftMax activation function, as shown in Figure 5.

#### 3.3.2. Sequential Seq_CNN Model

**Figure 6:**
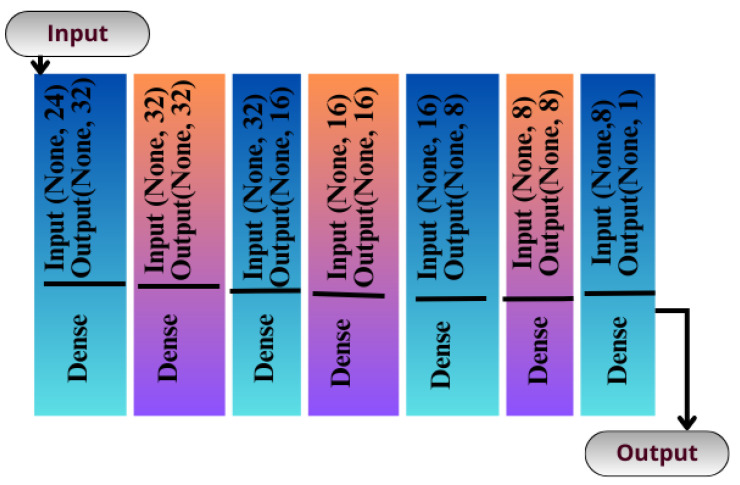
Seq-CNN model architecture.

The CNN can be instantiated as a sequential model because it is the simplest way of building artificial networks, consisting of layers stacked upon each other. Unlike the functional API, this method does not provide many features. It is not designed to create a complex artificial network with multiple paths. Rather, it is designed to create an artificial network with a single flow of data. There is no explicit input layer in this type of network, but it is integrated into another layer of processing. An illustration of the structure of this network can be seen in Figure 6 which consists of eight layers.

## 4. The Experimental Setting

A Keras package (2.2.4) with TensorFlow (1.13.1) backend was used to build the deep learning-related models. The system configuration was NVidia, core i7, Windows 11, and 64 GB of RAM. The configuration of the experiments conducted is represented in Table 4.

### Evaluation Metrics

ML models were evaluated with each set of important features based on how well they did in testing. An assessment of a classifier’s performance using the confusion matrix is conducted on a dataset where its classifier’s real values are already known. The terms are described in the following: true positives (TPs)—this indicates that CKD is expected to occur. True negatives (TN)—this indicates that CKD is unlikely to occur. False positive (FP)—this indicates that CKD is expected to occur, but the prediction is false (called type I error). False negative (FN)—in this case, CKD is not expected (called a Type II error). Using the formula given below in the table, classification reports are constructed using the following measures of sensitivity, specificity, precision, and F1 score. A confusion matrix is a table-like structure used to describe or evaluate a classifier’s performance. This matrix, despite its confusing terminology, is generally easy to understand and simple to use. The ROC curve is used as a metric to evaluate the performance of binary classification problems. Its function is to calculate the difference between true positive and false positive rates.

(10) $$Accuracy=\frac{\left(TP+TN\right)}{\left(TP+FP+FN+TN\right)}$$

(11) $$Sensitivity=\frac{TP}{\left(TP+FN\right)}$$

(12) $$Specificity=\frac{TP}{\left(TP+FP\right)}$$

(13) $$FDR=\frac{FP}{\left(FP+TP\right)}$$

(14) $$F-score=\frac{\left(2*TP\right)}{\left(2*TP+FP+FN\right)}$$

## 5. Experimental Results and Discussion

The performance of the API_CNN and Seq_CNN models will be explored in this section. This is followed by a formal comparison with three general machine learning models. Finally, the majority of relevant work for CKD detection will be summarized.

### 5.1. Deep Learning Performance

The performance of the API_CNN and Seq_CNN models is shown in Table 5.

**Figure 7:**
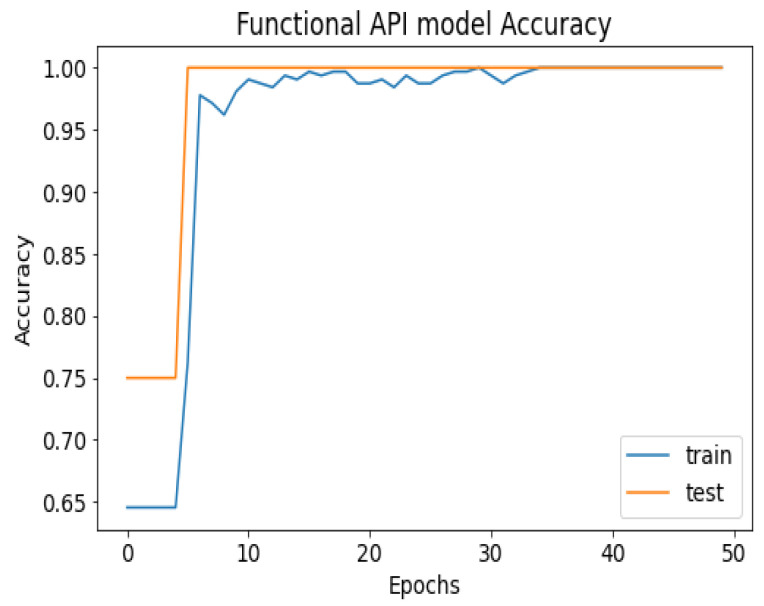
API_CNN model accuracy.

**Figure 8:**
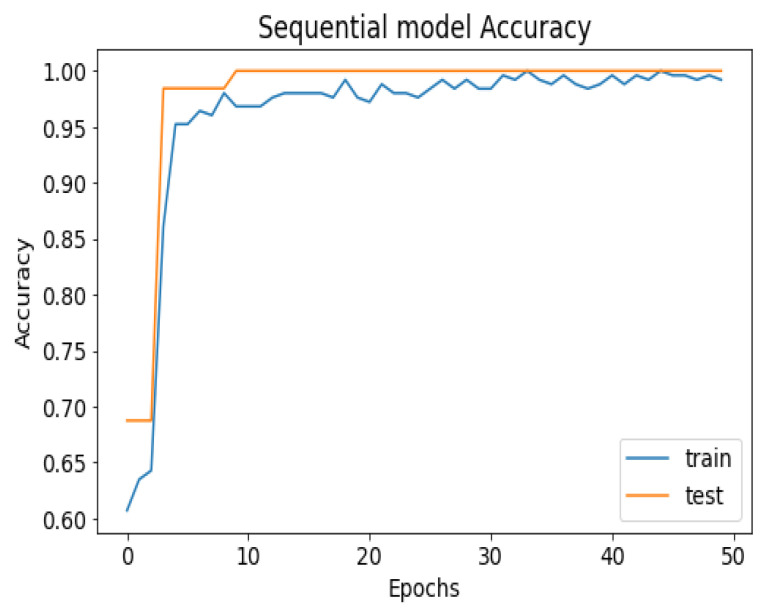
Seq_CNN model accuracy.

**Figure 9:**
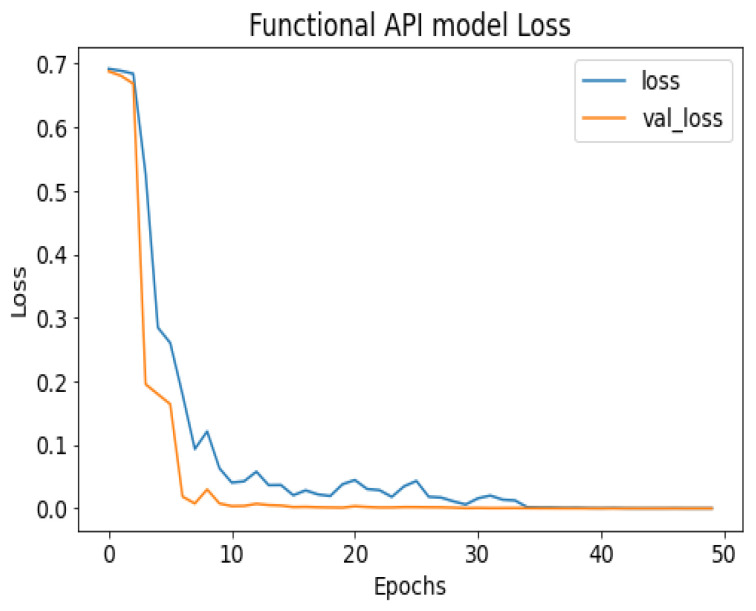
API_CNN model loss.

**Figure 10:**
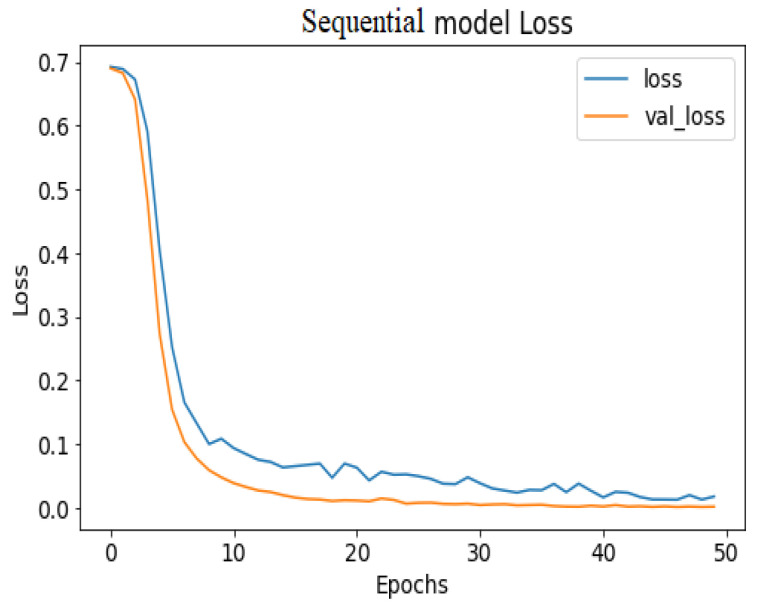
Seq_CNN model loss.

API_CNN was trained on 50 epochs of data and achieved a level of accuracy of 100%. Using the test data, the model achieved an accuracy level of 97.5% (Shown in Figure 7), and the cost function in the test data was 0.03. Additionally, the model achieved a precision level of 97% and a recall level of 92.9%. In training on 50 epochs of data, Seq_CNN achieved a level of accuracy of 99.7% (shown in Figure 8). In the test data, the model achieved a level of accuracy of 98.8%, and the cost function in the test data was 2%. On the precision scale, the model achieved 97.3%, and on the recall scale 100%. Figure 9 and Figure 10 show the proposed CNNs’ training and validation losses.

**Figure 11:**
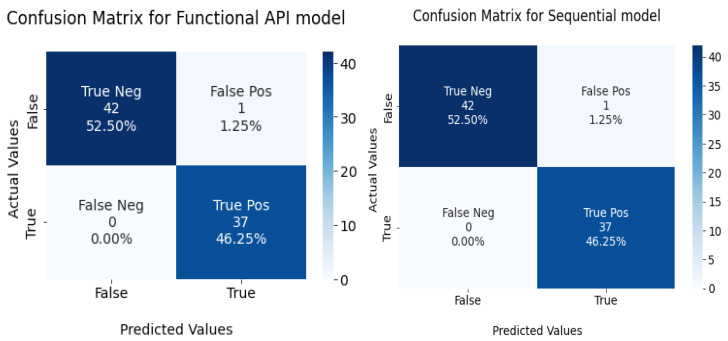
Confusion matrix for functional API and sequential model in the test set.

The confusion matrix for the devised models is given in Figure 11.

**Figure 12:**
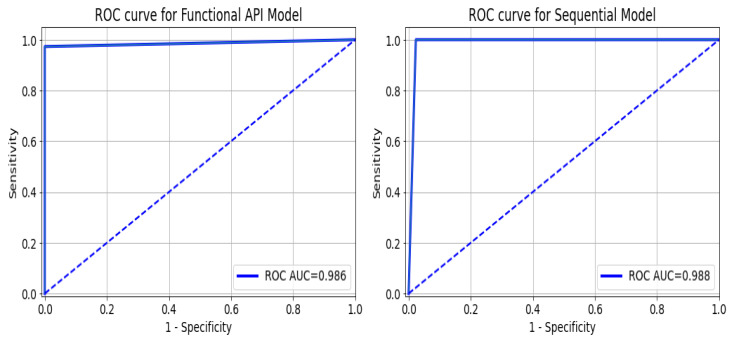
ROC curve for functional API_CNN and Seq_CNN in the test set.

As a summary of the ROC curve, the Area Under the Curve (AUC) measures the ability of a classifier to distinguish between classes. As the AUC moves to the left, the model is considered to be performing better in determining whether a class is positive or negative. As shown in Figure 12, the API_CNN model has an area under the curve of 97.5% while the Seq_CNN model has an area under the curve of 98.8%.

### 5.2. Performance Comparison with Machine Learning Algorithms

**Figure 13:**
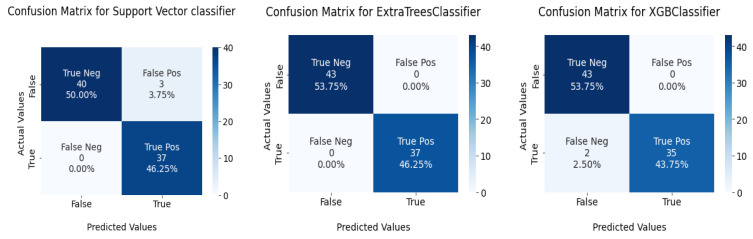
Confusion matrix for ML classifiers: from left to right SVC, ExtraTree, and XGBOOST.

**Figure 14:**
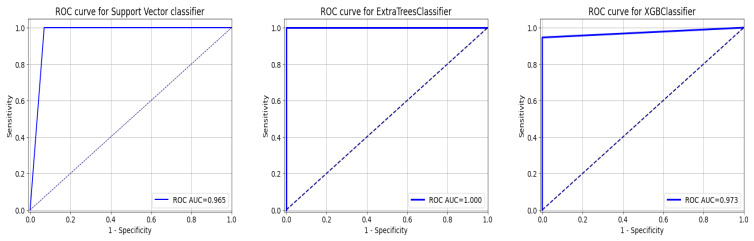
ROC Curve for ML Classifiers: from left to right SVC, ExtraTree, and XGBOOST.

This section tests the effectiveness of the devised preprocessing pipeline and feature selection using 3 classifiers namely; support vector, ExtraTrees, and XGBoost classifiers. It is evident from Table 6 and Table 7 that the accuracy of the fundamental machine learning classifiers has improved. ExtraTree is the most accurate prediction model for chronic kidney disease, with an accuracy rate of 100%. Figure 13 illustrates the confusion matrix for the models and AUC is given in Figure 14.

### 5.3. Performance Comparison with Related Work

The literature contains a significant amount of research for the prediction of chronic kidney disease (CKD), as shown in Table 8. We compare the methods of our system with some related work to evaluate their performance. Comparative research shows that the proposed method can detect CKDs with greater accuracy than in previous work.

### 5.4. Discussion

The objective of this study is to use machine learning to detect CKD in its earliest stages by dealing with the complexities associated with medical data. Additionally, we developed a lighter snake-optimized feature selection algorithm and customized CNN model for CKD detection which potentially reduced diagnostic time and cost. The problem of feature selection (FS) involves initializing a solution using a binary vector. The length of this vector corresponds to the dimensionality of the problem, with each bit representing a feature in a dataset. The values of the elements are either ’0’ or ’1’, where ’0’ indicates that a feature is not selected, and ’1’ indicates that a feature is selected. In cases where all features are selected, the search algorithm’s runtime becomes exponentially long. Consequently, in each iteration, the SO algorithm intelligently navigates towards improved search areas by leveraging the best solution obtained from the collected dataset.

Figure 7 shows the training and test results after 50 iterations of training. It can be observed that initially, the test accuracy is higher than the training accuracy on average for the first 9 iterations, but as the epoch progresses, the training and testing accuracy become more similar. After training the first eight epochs, the training and test became close to 90%. As the epoch progressed, the accuracy of the train and test increased. As shown in Table 4, other model evaluation metrics have been generated once training has been completed.

The 100% training accuracy of the API_CNN model (Figure 8 ) shows a low bias, i.e., it can learn training data correctly; compared to a test accuracy of 97.8% indicates that it can also generalize well to unknown data with 3.5% test loss. The Seq_CNN architecture is better suited to this use case due to its low computational complexity (model architecture is given in Figure 6) without suffering from an overfitting problem. Furthermore, based on the results shown in Table 4, it can be concluded that a precision of 97.3% indicates that the model correctly predicted the cases of CKD. Thus, the model has an accurate classification of the CKD data in 97.3% of the predicted patients. A recall of 100% means that the model successfully identified 100% or all of the actual CKD cases. Since there is no class imbalance in the dataset, an F1 score of 97% indicates strong model performance. The model has a specificity of 92.9%, which means that it successfully identified 92.9% of actual cases of non-CKD. A training accuracy of 98.8% for a sequential model means that the model has a low bias, i.e., it has learned the training data effectively. The model can be effectively applied to new data, as shown by the test accuracy of 99.7%. However, CNN models had an overfitting issue because of the architecture’s relative complexity, which hindered their generalization ability. On the CKD experimental data, the devised sequential CNN has made considerable progress in fitting the data (test loss of 2.4%) while also being able to generalize well with 97.3%, and 100% for precision and recall, respectively. The efficiency of the designed data preprocessing stage and snake-optimized feature selection algorithm is also evaluated using a formal representation over three machine learning algorithms (given in Table 6 and Table 7). To address their classification robustness, the cross-validation approach is used. Despite the fact that medical records commonly contain flaws that could cause potential data leakage, the accuracy of the classification using the data preparation pipeline and the snake-optimized feature selection algorithm has reached 100% using the ExtraTree classifier. Table 8 illustrates the accuracy of the model developed compared to a variety of previous approaches that used the same data set. In this evaluation, system models are compared to the most successful initiatives in the detection phenomenon of CKD in terms of testing/ training accuracy, and the utilization of optimization algorithms. Compared to other methods, the suggested method offers improved effectiveness and generalizability while selecting the optimal features using a natural-inspired algorithm. The time complexity of the proposed architecture is influenced by two main factors: the feature optimization process and the number of deep learning architectures used for classification. The time complexity of the framework, when employing 2-customized architectures, can be represented as O(2N), with *N* being the number of networks used. Since this study uses two DNN networks (N=2), the computational complexity is O(4). Additionally, the feature optimization process contributes a computational complexity of O(VK), where *V* denotes the number of running experiments and *K* represents the number of iterations for the optimization algorithm [48].

Therefore, the overall computational complexity of the proposed network is given by the sum of the complexities: O(2N)+O(VK). Consequently, it’s important to acknowledge certain limitations of this study. Firstly, data pre-processing requires a significant amount of time. However, it should be noted that the CKD parameters for our categorization networks are automatically selected and reduced using the SO algorithm, which provides an optimal composite feature set to the networks. Thus, the proposed CKD diagnostic method faces similar computational and storage challenges as existing methods.

## 6. Conclusions and Future Work

In this paper, a novel optimized learning technique for CKD detection is suggested. The main objective of the study is to suggest an efficient system that achieves maximum accuracy compared to other existing CKD detection techniques using a snake optimization feature selection algorithm. The suggested model was tested in 400 cases, which were split into CKD and non-CKD instances. On testing 20% of the total epochs, the suggested method attained an accuracy of 99.7% with 100% sensitivity. The proposed method for CKD detection is effective and reliable compared to other earlier methods. The devised system may eventually be implemented for real-time detection and forecasting. The research can also be expanded by including domain expertise in outlier detection, which will increase the robustness of the suggested approach. Additionally, deep learning algorithms can be used to provide precise medicine by identifying subgroups of CKD patients most likely to respond to specific treatments. This process could lead to more personalized and effective treatment for CKD patients. In addition, it will be possible to investigate the performance of superior nonlinear neural networks driven by artificial intelligence such as SOFNN-HPS and GK-ARFNN to predict the treatment processes of CKD.

## Figures and Tables

**Table 1:** State-of-the-art CKD detection methods.

Ref.	Method	Pros	Cons
Alsekait et al. [31]	A Pretrained deep learning models including LSTM, CNN, and GRU are integrated with SVMs as a metalearner models.	- A high degree of classification accuracy is achieved by the use of pre-trained deep - The utilization of four methods of feature selection for CKD detection.	- A significant calculation is needed to optimize the pretrained network. - The accuracy of the models obtained for the training or testing phases has not been formalized.
Hassan et al. [33]	For the detection of Kidney Disease, a variety of machine-learning algorithms are used. In addition, CKD features were selected using the XGBoost algorithm.	The devised feature selection algorithm enhances the classification task in comparison to a manual selection process.	The model has been trained using unbalanced blurred data which may produce biased results.
Vasquez et al. [34]	A five layers Neural Network based classifier for CKD detection. An adequate explanation was provided to support the precision of the forecast using CBR example.	The random forest classifier was used to select the features relevant to CKD based on the information provided.	Since the nearest neighbor method is used to represent each case, the performance of the CBR process is influenced by the number of features/variables present in the case.
Sawhney et al. [35]	Multi-Layer Perceptron based deep neural networks are used to detect CKD in patients.	A high level of accuracy has been achieved in the detection of kidney disease.	- The model has been trained using unbalanced blurred data which may produce biased results. -The most relevant features are selected based on a classification technique which takes considerable time to process.
Pal et al. [36]	Majority voting of three machine learning algorithms including RF, SVM, and ANN.	Three different patterns of chronic kidney disease have been examined.	The most relevant features patterns are selected manually which takes considerable time to process.
Gazi et al. [37]	Three ML techniques including KNN, LR, and DT.	A high level of accuracy has been achieved in the detection of kidney disease.	A correlation coefficient was calculated for only 14 of the attributes examined by the authors.
Chittora et al. [38]	Random Forest, SVM, and DT, KNN	Three different methods of feature selection were applied as part of the important feature selection process.	The most relevant features patterns are selected manually which takes considerable time to process.

**Table 2:** CKD dataset description.

Seq	Column Name	Count	Type	Seq	Column Name	Count	Type
0	id	400	int64	13	sod	313	float64
1	age	391	float64	14	pot	312	float64
2	bp	388	float64	15	hemo	348	float64
3	sg	353	float64	16	pcv	330	object
4	al	354	float64	17	wc	295	object
5	su	351	float64	18	rc	270	object
6	rbc	248	object	19	htn	398	object
7	pc	335	object	20	dm	398	object
8	pcc	396	object	21	cad	398	object
9	ba	396	object	22	appet	399	object
10	bgr	356	float64	23	pe	399	object
11	bu	381	float64	24	ane	399	object
12	sc	383	float64	25	classification	400	object

**Table 3:** CKD dataset statistics.

variables(#)	25	Duplicate rows	0
observations(#)	400	Variable types	Numeric (13) Categorical (7) Boolean (5)
Missing cells	1012		

**Table 4:** Overview of the experiment parameters.

Configuration Parameters	Specification	Configuration Parameters	Specification
Input Data	[numerical, categorical]	Dataset Training/testing	80% training, 20% testing
Data Shuffling	Yes (Random)	Classifier	SVM, XGBoost, ExtraTreesClassifier, API-CNN, Seq-CNN
Class imbalance	Yes [SMOTE]	Outlier Detection	YES [‘age’,‘hemo’, ‘pcv’,‘rc’,‘sg’]
Input Data Size	CKD: 150. Not-CKD: 250.	features selection	snake optimization
Missing values	Yes [replace(np.nan,‘?’), compensation]	batch_size	10
Encoding Categorical Variables	Yes [0, 1]	duplicate values	[Yes, no]
Feature correlation	Yes [00-0.19 ‘very weak’ 20-0.39 “weak” 40-0.59 “moderate” 60-0.79 “strong” 80-1.0 “very strong”]	Epochs number	50
Loss	‘Binary_crossentropy’	Optimizer	Adam

**Table 5:** Overview of API_CNN and Seq_CNN models performance.

	Training Accuracy	Test Accuracy	Test Loss	Precision	Recall	F1-Score
API_CNN	100%	97.5%	3.5%	97.3%	92.9%	97%
Seq_CNN	98.8%	99.7%		97.3%	1.00%	99%

**Table 6:** Comparison with previous methods for CKD detection.

	Training Accuracy	Test Accuracy	Precision	Recall	F1-Score
SVC	96.25%	92.5%	100%	93%	96%
ExtraTrees	100%	100%	100%	100%	100%
XGBoost	100%	97.5%	96%	100%	98%

**Table 7:** The performance metric of the proposed method after being subjected to 10-fold cross-validation with ML classifiers.

	Fold-1	Fold-2	Fold-3	Fold-4	Fold-5	Fold-6	Fold-7	Fold-8	Fold-9	Fold-10
SVC	97.5%	96.25%	100%	92.5%	92.5%	92.5%	100%	95%	97.5%	95%
ExtraTree	97.5%	100%	100%	100%	97.5%	100%	100%	100%	95%	100%
XGBoost	97%	100%	100%	95%	97.5%	100%	100%	100%	92.5%	100%

**Table 8:** CKD-SO performance comparison with state-of-the-art CKD detection methods.

	Classifier(s)	Training Accuracy (%)	Testing Accuracy (%)	AUC (%)	Optimization
[27]	Ensemble of KELM, DBN, and CNN-GRU models	96.91%	N/A	99.72%	×
[38]	Random forest (RF), Support Vector Machine (SVM), and Decision Tree(DT), KNN, with random forest is the best	100%	N/A	N/A	×
[23]	Random forest, DT, SVM, and k-nearest neighbors (KNN).	100% 99.34% 98.33% 97.3%	N/A	N/A	×
[13]	Convolutional autoencoder	71%	N/A	N/A	×
[45]	CNN, Bi-LSTM	89% 87%	N/A	95.7%, 93.9%	×
[46]	DNN	100%	N/A	100%	×
[32]	Ant Colony-Based Optimization	95%	N/A	N/A	√
[47]	Logistic regression (LR), Random forest (RF), Support vector machine (SVM), K-nearest neighbors (KNN), EXtreme gradient boosting (XGB)	99.1% 98.1% 98.9% 94.9% 98.3%	N/A	100 99.3% 99.9% 98.1% 99.5%	×
[28]	Bagging of Logistic Regression (LR), Decision Tree (DT), and Support Vector Machine (SVM)	97.23%	N/A	N/A	√
[32]	D-ACO framework based on Genetic algorithms (GA), Adaptive classification (AC), Particle swarm optimization (PSO).	87.50% 85% 75%	N/A	N/A	√
CKD-SO	Snake-based optimization using SVM Extra Trees XGB API_CNN Seq_CNN	96.25% 100% 100% 100% 98.8%	96.25% 100% 97.5% 97.5% 99.7%	96.5% 100% 97.3% 97.5% 98.8%	√

## Data Availability

The dataset is publically available at ‘https://archive.ics.uci.edu/dataset/336/chronic+kidney+disease’.

## Abbreviations

In this manuscript, the following abbreviations are used:

CKD	Chronic kidney disease
CNN	Convolutional neural network
SOA	Snake optimization algorithm
DL	Deep learning
API-CNN	Functional API CNN model
Seq_CNN	Sequential CNN model
SVM	Support Vector Machine
ExtraTreesClassifie	Extremely Randomized Trees Classifier
XGBoost	Extreme Gradient Boosting
LOF	Local outlier factor
PSO	Particle swarm optimization
GA	Genetic algorithms
AC	Adaptive classification
SMOTE	Synthetic Minority Over-sampling Technique
CBR	Case-based reasoning
AUC	Area Under the Curve

## References

[1] ElSayed N.A., Aleppo G., Aroda V.R., Bannuru R.R., Brown F.M., Bruemmer D., Collins B.S., Hilliard M.E., Isaacs D., Johnson E.L. (2023). 11. Chronic Kidney Disease and Risk Management: Standards of Care in Diabetes—2023. Diabetes Care.

[2] Smidtslund P., Jansson Sigfrids F., Ylinen A., Elonen N., Harjutsalo V., Groop P.H., Thorn L.M. (2023). Prognosis After First-Ever Myocardial Infarction in Type 1 Diabetes Is Strongly Affected by Chronic Kidney Disease. Diabetes Care.

[3] WHO (2021). WHO Global Report on Trends in Prevalence of Tobacco Use 2000–2025.

[4] Gumaei A., Ismail W.N., Hassan M.R., Hassan M.M., Mohamed E., Alelaiwi A., Fortino G. (2022). A decision-level fusion method for COVID-19 patient health prediction. Big Data Res..

[5] Ismail W.N., Hassan M.M., Alsalamah H.A., Fortino G. (2020). CNN-based health model for regular health factors analysis in internet-of-medical things environment. IEEE Access.

[6] Almansour N.A., Syed H.F., Khayat N.R., Altheeb R.K., Juri R.E., Alhiyafi J., Alrashed S., Olatunji S.O. (2019). Neural network and support vector machine for the prediction of chronic kidney disease: A comparative study. Comput. Biol. Med..

[7] Ismail W.N., Rajeena PP F., Ali M.A. (2022). MULTforAD: Multimodal MRI Neuroimaging for Alzheimer’s Disease Detection Based on a 3D Convolution Model. Electronics.

[8] Ismail W.N., Hassan M.M., Alsalamah H.A. (2019). Context-enriched regular human behavioral pattern detection from body sensors data. IEEE Access.

[9] Serte S., Serener A., Al-Turjman F. (2022). Deep learning in medical imaging: A brief review. Trans. Emerg. Telecommun. Technol..

[10] Bengio Y., Courville A. (2013). Deep learning of representations. Handbook on Neural Information Processing.

[11] Acharya U.R., Meiburger K.M., Koh J.E.W., Hagiwara Y., Oh S.L., Leong S.S., Ciaccio E.J., Wong J.H.D., Shah M.N.M., Molinari F. (2020). Automated detection of chronic kidney disease using higher-order features and elongated quinary patterns from B-mode ultrasound images. Neural Comput. Appl..

[12] Nithya A., Appathurai A., Venkatadri N., Ramji D., Palagan C.A. (2020). Kidney disease detection and segmentation using artificial neural network and multi-kernel k-means clustering for ultrasound images. Measurement.

[13] Drall S., Drall G.S., Singh S., Naib B.B. (2018). Chronic kidney disease prediction using machine learning: A new approach. Int. J. Manag..

[14] Bhaskar N., Suchetha M. (2021). A computationally efficient correlational neural network for automated prediction of chronic kidney disease. IRBM.

[15] Bevilacqua V., Brunetti A., Cascarano G.D., Palmieri F., Guerriero A., Moschetta M. A deep learning approach for the automatic detection and segmentation in autosomal dominant polycystic kidney disease based on magnetic resonance images. Proceedings of the International Conference on Intelligent Computing.

[16] Ismail W.N., PP F.R., Ali M.A. (2023). A Meta-Heuristic Multi-Objective Optimization Method for Alzheimer’s Disease Detection Based on Multi-Modal Data. Mathematics.

[17] Ismail W.N., Alsalamah H.A., Hassan M.M., Mohamed E. (2023). AUTO-HAR: An adaptive human activity recognition framework using an automated CNN architecture design. Heliyon.

[18] Hashim F.A., Hussien A.G. (2022). Snake Optimizer: A novel meta-heuristic optimization algorithm. Knowl.-Based Syst..

[19] Shahamatnia E., Ebadzadeh M.M. (2011). Application of particle swarm optimization and snake model hybrid on medical imaging. Proceedings of the 2011 IEEE Third International Workshop On Computational Intelligence in Medical Imaging.

[20] Swain D., Mehta U., Bhatt A., Patel H., Patel K., Mehta D., Acharya B., Gerogiannis V.C., Kanavos A., Manika S. (2023). A Robust Chronic Kidney Disease Classifier Using Machine Learning. Electronics.

[21] Kotanko P., Nadkarni G.N. (2023). Advances in Chronic Kidney Disease Lead Editorial Outlining the Future of Artificial Intelligence/Machine Learning in Nephrology. Adv. Kidney Dis. Health.

[22] Krishnamurthy S., Ks K., Dovgan E., Luštrek M., Gradišek Piletič B., Srinivasan K., Li Y.C., Gradišek A., Syed-Abdul S. (2021). Machine learning prediction models for chronic kidney disease using national health insurance claim data in Taiwan. Healthcare.

[23] Senan E.M., Al-Adhaileh M.H., Alsaade F.W., Aldhyani T.H., Alqarni A.A., Alsharif N., Uddin M.I., Alahmadi A.H., Jadhav M.E., Alzahrani M.Y. (2021). Diagnosis of chronic kidney disease using effective classification algorithms and recursive feature elimination techniques. J. Healthc. Eng..

[24] Tusar M.T.H.K., Islam M.T., Raju F.I. Detecting Chronic Kidney Disease (CKD) at the Initial Stage: A Novel Hybrid Feature-selection Method and Robust Data Preparation Pipeline for Different ML Techniques. Proceedings of the 2022 5th International Conference on Computing and Informatics (ICCI).

[25] Debal D.A., Sitote T.M. (2022). Chronic kidney disease prediction using machine learning techniques. J. Big Data.

[26] Yashfi S.Y., Islam M.A., Sakib N., Islam T., Shahbaaz M., Pantho S.S. Risk prediction of chronic kidney disease using machine learning algorithms. Proceedings of the 2020 11th International Conference on Computing, Communication and Networking Technologies (ICCCNT).

[27] Alsuhibany S.A., Abdel-Khalek S., Algarni A., Fayomi A., Gupta D., Kumar V., Mansour R.F. (2021). Ensemble of Deep Learning Based Clinical Decision Support System for Chronic Kidney Disease Diagnosis in Medical Internet of Things Environment. Comput. Intell. Neurosci..

[28] Pal S. (2022). Chronic Kidney Disease Prediction Using Machine Learning Techniques. Biomed. Mater. Devices.

[29] Zhou H., Zhang Y., Duan W., Zhao H. (2020). Nonlinear systems modelling based on self-organizing fuzzy neural network with hierarchical pruning scheme. Appl. Soft. Comput..

[30] Zhou H., Li Y., Zhang Q., Xu H., Su Y. (2022). Soft-sensing of effluent total phosphorus using adaptive recurrent fuzzy neural network with Gustafson-Kessel clustering. Expert Syst. Appl..

[31] Alsekait D.M., Saleh H., Gabralla L.A., Alnowaiser K., El-Sappagh S., Sahal R., El-Rashidy N. (2023). Toward Comprehensive Chronic Kidney Disease Prediction Based on Ensemble Deep Learning Models. Appl. Sci..

[32] Elhoseny M., Shankar K., Uthayakumar J. (2019). Intelligent diagnostic prediction and classification system for chronic kidney disease. Sci. Rep..

[33] Hassan M.M., Hassan M.M., Mollick S., Khan M.A.R., Yasmin F., Bairagi A.K., Raihan M., Arif S.A., Rahman A. (2023). A Comparative Study, Prediction and Development of Chronic Kidney Disease Using Machine Learning on Patients Clinical Records. Hum.-Centric Intell. Syst..

[34] Vásquez-Morales G.R., Martinez-Monterrubio S.M., Moreno-Ger P., Recio-Garcia J.A. (2019). Explainable prediction of chronic renal disease in the colombian population using neural networks and case-based reasoning. IEEE Access.

[35] Sawhney R., Malik A., Sharma S., Narayan V. (2023). A comparative assessment of artificial intelligence models used for early prediction and evaluation of chronic kidney disease. Decis. Anal. J..

[36] Pal S. (2023). Prediction for chronic kidney disease by categorical and non_categorical attributes using different machine learning algorithms. Multimed. Tools Appl..

[37] Ifraz G.M., Rashid M.H., Tazin T., Bourouis S., Khan M.M. (2021). Comparative analysis for prediction of kidney disease using intelligent machine learning methods. Comput. Math. Methods Med..

[38] Chittora P., Chaurasia S., Chakrabarti P., Kumawat G., Chakrabarti T., Leonowicz Z., Jasiński M., Jasiński Ł., Gono R., Jasińska E. (2021). Prediction of chronic kidney disease-a machine learning perspective. IEEE Access.

[39] Kotsiantis S., Kanellopoulos D., Pintelas P. (2006). Handling imbalanced datasets: A review. GESTS Int. Trans. Comput. Sci. Eng..

[40] Smiti A. (2020). A critical overview of outlier detection methods. Comput. Sci. Rev..

[41] Kaur H., Pannu H.S., Malhi A.K. (2019). A systematic review on imbalanced data challenges in machine learning: Applications and solutions. ACM Comput. Surv. (CSUR).

[42] Tyagi S., Mittal S. (2020). Sampling approaches for imbalanced data classification problem in machine learning. Proceedings of the ICRIC 2019.

[43] Mohammed A.J., Hassan M.M., Kadir D.H. (2020). Improving classification performance for a novel imbalanced medical dataset using SMOTE method. Int. J. Adv. Trends Comput. Sci. Eng..

[44] Parmar C., Barry J.D., Hosny A., Quackenbush J., Aerts H.J. (2018). Data analysis strategies in medical imaging. Clin. Cancer Res..

[45] Makino M., Yoshimoto R., Ono M., Itoko T., Katsuki T., Koseki A., Kudo M., Haida K., Kuroda J., Yanagiya R. (2019). Artificial intelligence predicts the progression of diabetic kidney disease using big data machine learning. Sci. Rep..

[46] Singh V., Asari V.K., Rajasekaran R. (2022). A Deep Neural Network for Early Detection and Prediction of Chronic Kidney Disease. Diagnostics.

[47] Hossain M.M., Swarna R.A., Mostafiz R., Shaha P., Pinky L.Y., Rahman M.M., Rahman W., Hossain M.S., Hossain M.E., Iqbal M.S. (2022). Analysis of the performance of feature optimization techniques for the diagnosis of machine learning-based chronic kidney disease. Mach. Learn. Appl..

[48] Khurma R.A., Albashish D., Braik M., Alzaqebah A., Qasem A., Adwan O. (2023). An augmented Snake Optimizer for diseases and COVID-19 diagnosis. Biomed. Signal Process. Control..

